# Metagenomic Next-Generation Sequencing for the Microbiological Diagnosis of Abdominal Sepsis Patients

**DOI:** 10.3389/fmicb.2022.816631

**Published:** 2022-02-02

**Authors:** Dongkai Li, Wei Gai, Jiahui Zhang, Wei Cheng, Na Cui, Hao Wang

**Affiliations:** ^1^Department of Critical Care Medicine, Peking Union Medical College Hospital, Beijing, China; ^2^WillingMed Technology (Beijing) Co., Ltd, Beijing, China; ^3^Department of Critical Care Medicine, Beijing Jishuitan Hospital, Beijing, China

**Keywords:** intra-abdominal infection, sepsis, abdominal sepsis, mNGS, infection

## Abstract

**Objectives:**

For patients with intra-abdominal infection (IAI), the rapid and accurate identification of pathogens remains a challenge. Metagenomic next-generation sequencing (mNGS) is a novel technique for infectious diseases, but its application in IAI is limited. In this study, we compared the microbiological diagnostic ability of plasma mNGS with that of conventional peritoneal drainage (PD) culture in critical care settings.

**Methods:**

From January 2018 to December 2020, a prospective observational study was performed at a tertiary teaching hospital in China and data on 109 abdominal sepsis patients were collected. The pathogen detection performance of plasma mNGS and PD culture method were compared.

**Measurements and Results:**

Ninety-two positive cases detected on PD culture, while plasma mNGS detected 61 positive cases. Forty-five patients (44.0%) had at least one matched pair of plasma mNGS and PD culture results. Compared with PD culture, the plasma mNGS was more rapid (27.1 ± 4.0 vs. 68.9 ± 22.3 h, *p* < 0.05). The patients received initial antibiotic treatment matched with mNGS detection showed better clinical outcomes.

**Conclusion:**

For abdominal sepsis patients, plasma mNGS can provide early, noninvasive, and rapid microbiological diagnosis. Compared with conventional PD smear, culture, and blood culture methods, plasma mNGS promote the rapid detection of pathogenic bacteria.

## Introduction

The abdomen cavity is the second most frequent source of sepsis and is associated with increased morbidity and mortality (Sartelli et al., 2014; Ross et al., 2018; Martin-Loeches et al., 2019; Sartelli, 2020), thus rapid diagnosis and initial treatment are necessary in clinical settings. However, initial empirical antibiotic therapy is often non-specific, which may lead to unnecessary exposure to broad-spectrum antibiotics, and needs to be adjusted according to the microorganism profile as soon as possible (Mazuski et al., 2002; Thorndike and Kollef, 2020). Furthermore, for sepsis patients with negative cultures, the rapid and accurate identification of pathogens remains a challenge (Tsuchiya et al., 2019). For intra-abdominal infection (IAI), culturing peritoneal drainage (PD) fluid is a common method of identifying the pathogen (Mazuski et al., 2002; Tsuchiya et al., 2019; Thorndike and Kollef, 2020). The percentage of positive PD culture in clinical settings varies between 19.5 and 63.7%, (Waele et al., 2014; Sim et al., 2020; Xiong and Rao, 2020) while the microbiological profiles (culture and susceptibility results) often require more than 72 h (Rajapaksha et al., 2019). Therefore, a novel culture-independent method is needed (Rhee et al., 2021).

Since the 2010s, metagenomic next-generation sequencing (mNGS) technology, which is based on nucleic acid sequencing and has the advantage of a high-throughput capacity and unbiased pathogen detection, has been applied and validated as a method of diagnosing infectious diseases (Lecuit and Eloit, 2015; Rossen and Friedrich, 2018; Gu et al., 2019). However, for patients with IAIs, relevant clinical studies investigating the performance of mNGS are limited. Therefore, this study was undertaken to compare the microbiological diagnostic ability of plasma mNGS with that of conventional peritoneal drainage culture for sepsis patients in critical care settings and to explore whether this promising and non-invasive tool for diagnosing infectious diseases can improve IAI care.

## Materials and Methods

### Patient Population and Study Design

This single-center prospective observational study evaluated data on abdominal sepsis patients admitted to Peking Union Medical College Hospital, which is a tertiary teaching hospital with 30 beds in the department of critical care medicine, between January 2018 and December 2020. Inclusion criteria were: (1) age ≥ 18 years; (2) ICU stay > 24 h; (3) proven or suspected severe intra-abdominal, nosocomial, or community-acquired infection (Sartelli et al., 2017); and (4) diagnosis with Sepsis 3.0 (Shankar-Hari et al., 2016). Exclusion criteria were: (1) incomplete clinical data, including microbiological data; (2) failure to acquire a sufficient sample for mNGS analysis during the first 24 h after ICU admission; (3) life expectancy of <24 h; and (4) failure to meet the inclusion criteria or obtain written consent. This study was approved by the institutional review board of Peking Union Medical College Hospital (approval number: JS-1170). Informed consent was obtained from all patients involved, and the study was registered at http://www.chictr.org.cn/ (identifier ChiCTR-ROC-17010750).

### Data Collection

In this study, patient demographics, clinical data (including the etiology), onset location, subsequent infection of the intra-abdominal region, Acute Physiology and Chronic Health Evaluation (APACHE) II score, Sequential Organ Failure Assessment (SOFA) score, and in-hospital mortality were recorded. During the first 24 h after ICU admission, peripheral blood samples were obtained for mNGS analysis, while intraperitoneal fluid samples were obtained at least three times for conventional rapid examination (Gram staining smear) and PD culture (both aerobic and anaerobic). The results of the mNGS were not relayed to the participants or their medical team. Only rapid examination and PD culture results were available as microbiological evidence for choosing therapeutic interventions for sepsis and IAI. Peripheral blood culture was also collected to diagnose the bloodstream infection and the time to initial positive detection and report was also recorded. Considering that viruses are not common pathogens of IAI, the detection of viruses by plasma mNGS was not included in this study. Culture and confirmation of species identification by PD fluid were performed at the central laboratory of the Clinical Laboratory Department, Peking Union Medical College Hospital, by matrix-assisted laser desorption/ionization time-of-flight mass spectrometry (Vitek MS; bioMérieux, Marcy l’Etoile, France).

### Metagenomic Next-Generation Sequencing and Data Analysis

After each sample was obtained, mNGS was performed *via* the following steps. *Sample collection and DNA extraction*: Whole-blood samples (2–5 ml) were collected in anticoagulation tubes. After centrifugation at 1,900×*g* for 10 min at 4°C, nucleic acids were extracted from the plasma and used for sequencing. Cell-free DNA was extracted from the plasma using PathoXtract Plasma Nucleic Acid Kit (WYXM03001S, Willingmed Corp., Beijing, China). DNA was eluted with 50 μl of nuclease-free water. *Library construction, sequencing, and data analysis*: libraries for NGS were prepared from cell-free DNA using the KAPA DNA HyperPrep Kit (KK8504, KAPA, Kapa Biosystems, Wilmington, MA, United States) in accordance with the manufacturer’s protocol. Sequencing of the libraries was performed on NextSeq 550Dx (Illumina), and at least 25 million sequencing reads were acquired for each sample. *Pipeline of bioinformatics analysis*: the genomic data of bacteria, fungi, viruses, parasites, archaea, and other pathogenic microorganisms were obtained from NCBI GenBank, and the clinical application-level reference database of pathogenic microorganisms was constructed through genomic filtering, screening, and validation. Sequencing data were processed using Pathogen Identification Sequencing (PIseq) Metagenomic Sequencing Data Management System V2.0 (Willingmed Corp.) automatically, and the detection report was generated. Quality control and evaluation of FASTQ format data obtained by sequencing were carried out, and low-quality or undetected sequences, sequences contaminated by splices, high-coverage repeats, and short read-length sequences were filtered to retain high-quality sequencing data. The high-quality sequencing data were compared with the human reference genome GRCH37 (hg19) by alignment software to remove the human host sequence and obtain clean data for use in the subsequent identification of pathogenic microorganisms. The clean data were aligned with the established reference database of pathogenic microorganisms to complete the annotation of pathogenic microorganism species, complete the final analysis, and obtain results on microorganism identification. For identification of pathogen, a RPTM value was used to identify positive pathogens, which defined as detected number of pathogen-specific reads per 10 million. Positive pathogens were required to meet a RPTM threshold ≥5 for bacteria and ≥5 for fungi. The sequencing data are available *via* BioProject accession number PRJNA749647.^1^

### Statistical Analysis

Measurement data were expressed as mean ± SD, median, and interquartile range, or proportions (absolute and relative frequencies), as appropriate. The Student’s *t*-test or Mann–Whitney U test was used to compare continuous variables; the *χ*^2^ test or Fisher’s exact test was used to compare categorical variables. Statistical analysis was performed by using IBM SPSS Statistics, version 24 (IBM Corp., Armonk, NY, United States). Differences with values of *p* < 0.05 were considered statistically significant.

## Results

### Patient Characteristics

**Figure 1:**
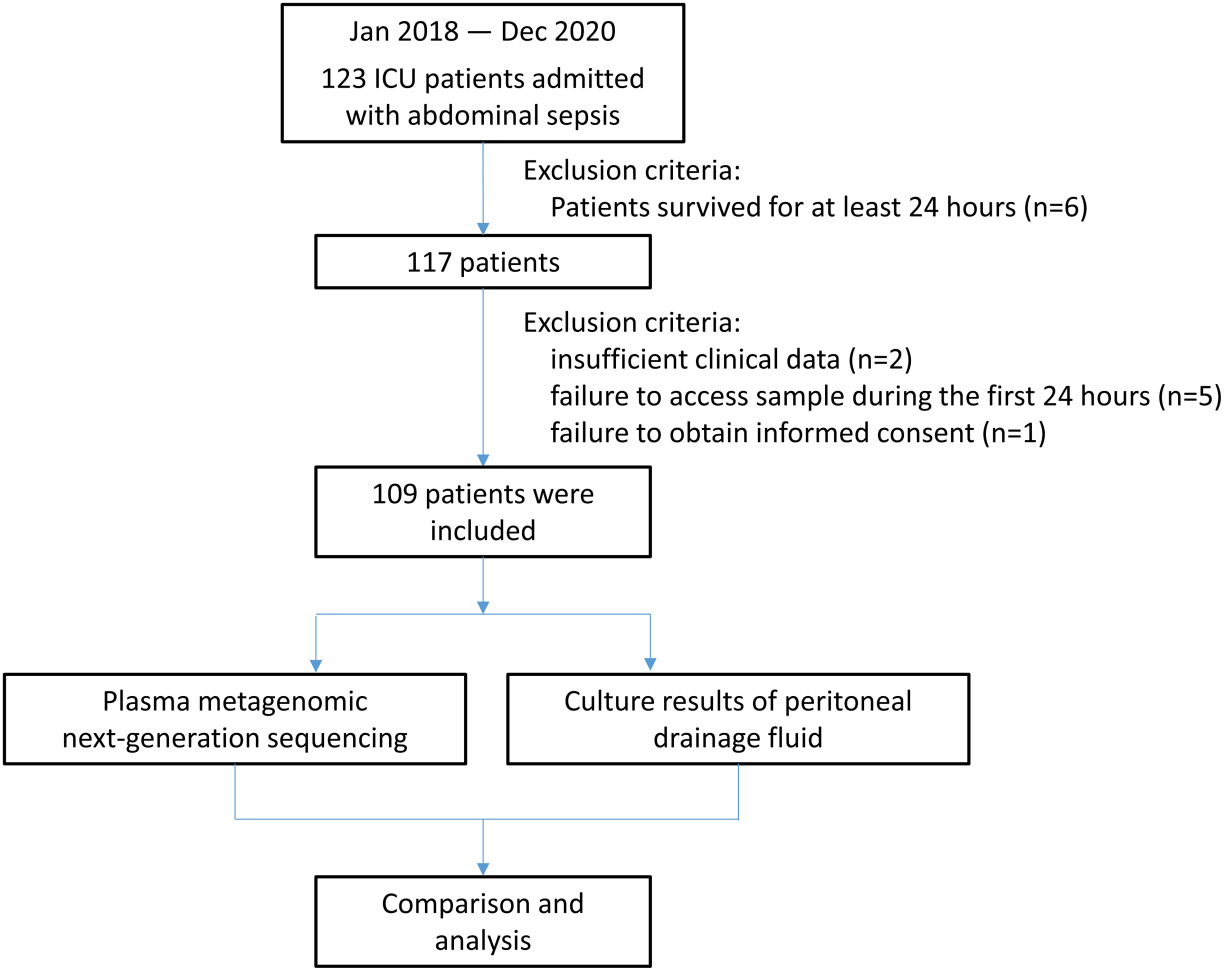
Flowchart of the prospective study.

During the study period, 123 patients were admitted to the ICU with abdominal sepsis, six of whom were excluded because they did not survive for at least 24 h, and eight patients were excluded owing to insufficient clinical data, as showed in Figure 1. Thus, 109 patients were enrolled in the final analysis (Table 1). No significant differences were identified between the plasma mNGS positive and negative groups in terms of age, sex, APACHE II score, SOFA score, infection etiology, onset location, or comorbidities. The etiological comparison showed that mNGS positive patients tended to be associated with intestinal perforation or obstruction compared with the mNGS negative (83.6% in mNGS positive vs. 70.8% in mNGS negative, *p* = 0.110), while the difference was statistically insignificant. The plasma mNGS positive patients also tend to be associated with a higher proportion of subsequent infections and hospital mortality (29.2% in mNGS positive vs. 13.1% in mNGS negative, *p* = 0.417), while the differences were also statistically insignificant.

**Table 1:** Demographic and clinical characteristics of study patients.

Characteristics	mNGS detection		*p*
	mNGS positive	mNGS negative	
*n*	61	48	
Age	62.4 ± 15.5	64.9 ± 16.7	0.423
Sex, male	42 (68.9%)	29 (60.4%)	0.359
APACHE II score	20.3 ± 7.1	20.0 ± 5.7	0.770
SOFA score	7.9 ± 2.2	7.4 ± 2.2	0.220
Comorbidities			
Hypertension	33 (54.1%)	24 (50.0%)	0.671
Diabetes mellitus	29 (47.5%)	22 (45.8%)	0.859
Chronic kidney dysfunction	11 (18.0%)	8 (16.7%)	0.852
Etiology			
Biliary tract disease, *n* (%)	3 (4.9%)	5 (10.4%)	0.470
Acute pancreatitis, *n* (%)	3 (4.9%)	1 (2.1%)	0.788
Intestinal perforation or obstruction, *n* (%)	51 (83.6%)	34 (70.8%)	0.110
Liver abscess, *n* (%)	0 (0.0%)	1 (2.1%)	0.904
Complicated appendicitis, *n* (%)	2 (3.3%)	2 (4.2%)	0.788
Others, *n* (%)	2 (3.3%)	5 (10.4%)	0.265
Onset location (*n*, %)			0.914
Community acquired	40 (65.6%)	31 (64.6%)	
Hospital acquired	21 (34.4%)	17 (35.4%)	
Subsequent infection (*n*, %)			
Bloodstream infection	8 (16.7%)	3 (4.9%)	0.389
Skin and soft tissue infections	5 (10.4%)	2 (3.3%)	0.647
Intestinal fistula	4 (8.3%)	3 (4.9%)	0.743
Hospital mortality (*n*, %)	14 (29.2%)	8 (13.1%)	0.417

APACHE II, acute physiology and chronic health evaluation II; SOFA, sequential organ failure assessment.

### Microbiological Diagnostic Performance of PD Culture and mNGS

**Figure 2:**
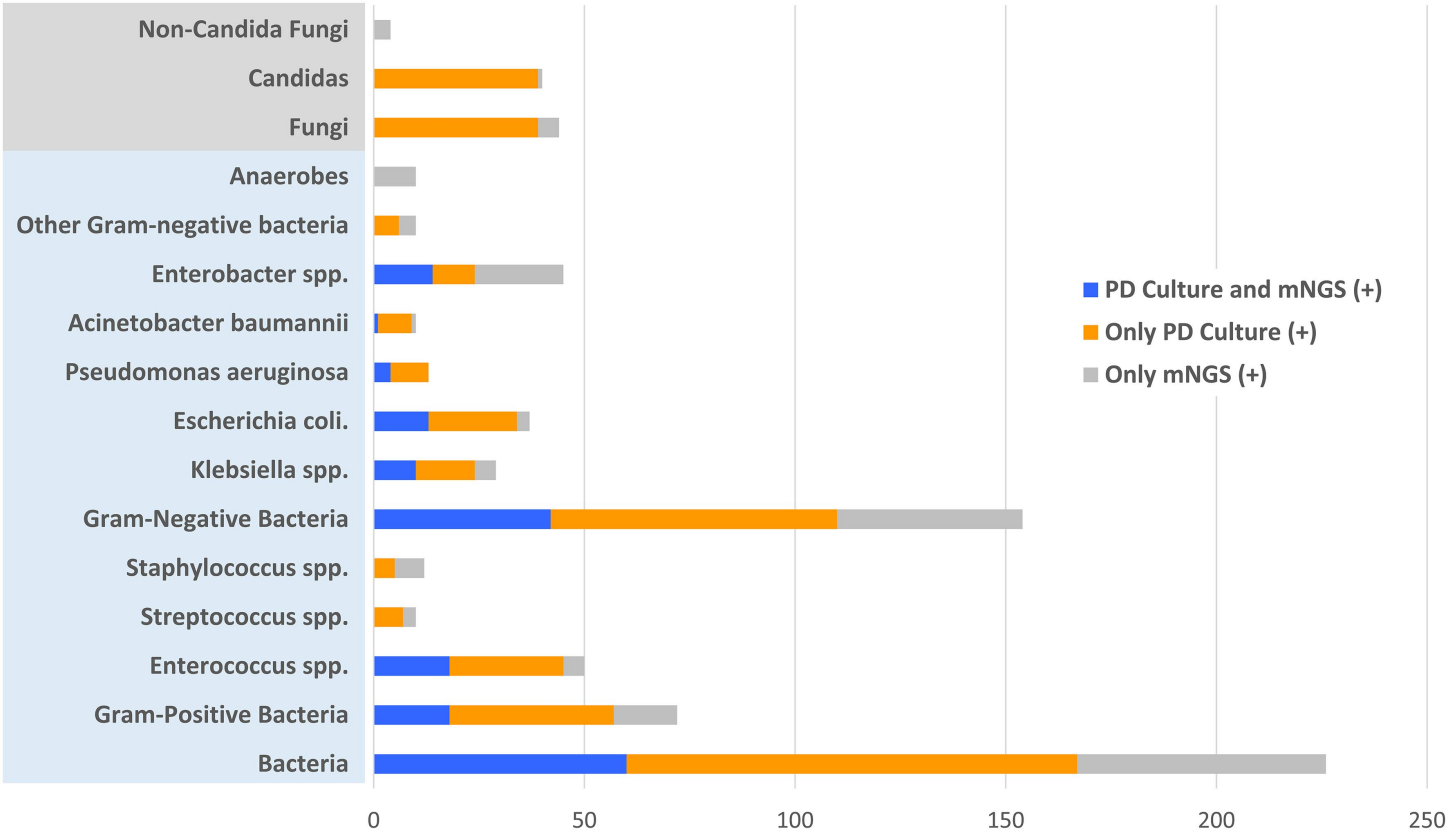
Distribution of positive and matched results of plasma metagenomic next-generation sequencing (mNGS) and peritoneal drainage (PD) culture according to the detected pathogenic strains. *X*-axis represents the counts of detected pathogenic strains.

All 109 patients received both conventional PD culture and plasma mNGS detection. Among them, PD culture detected 92 positive cases, while mNGS detected 61 positive cases. According to microbiological and clinical practice, the pathogens detected were classified and the results were compared between the two methods, as showed in Figure 2. According to the detected strains, there were fewer positive detection results from plasma mNGS than from PD culture for both bacterial (119 in plasma mNGS vs. 167 in PD culture, strains) and fungal pathogens (5 in mNGS vs. 39 in PD culture, strains). For bacterial pathogens, differences in the diagnostic methods were observed for Gram-positive [33 (28.0%) in mNGS vs. 59 (33.5%) in PD culture, *p* < 0.005] and Gram-negative pathogens [85 (72.0%) in plasma mNGS vs. 117 (66.5%) in PD culture, *p* < 0.005]. Comparing the ratio of Gram-negative to Gram-positive bacteria showed that plasma mNGS tended to detect more Gram-negative bacteria (Ratio of Gram−/Gram+: 2.57 vs. 1.98, *p* < 0.005). The contribution of plasma mNGS on pathogen detection (only mNGS positive: 59 bacteria strains and five fungi strains) was concentrated on the Gram-negative bacteria (44 strains only positive in mNGS) and among them the plasma mNGS method detected all 10 strains within the anaerobe category that the conventional PD culture method was unable to identify, as shown in Figure 2.

**Figure 3:**
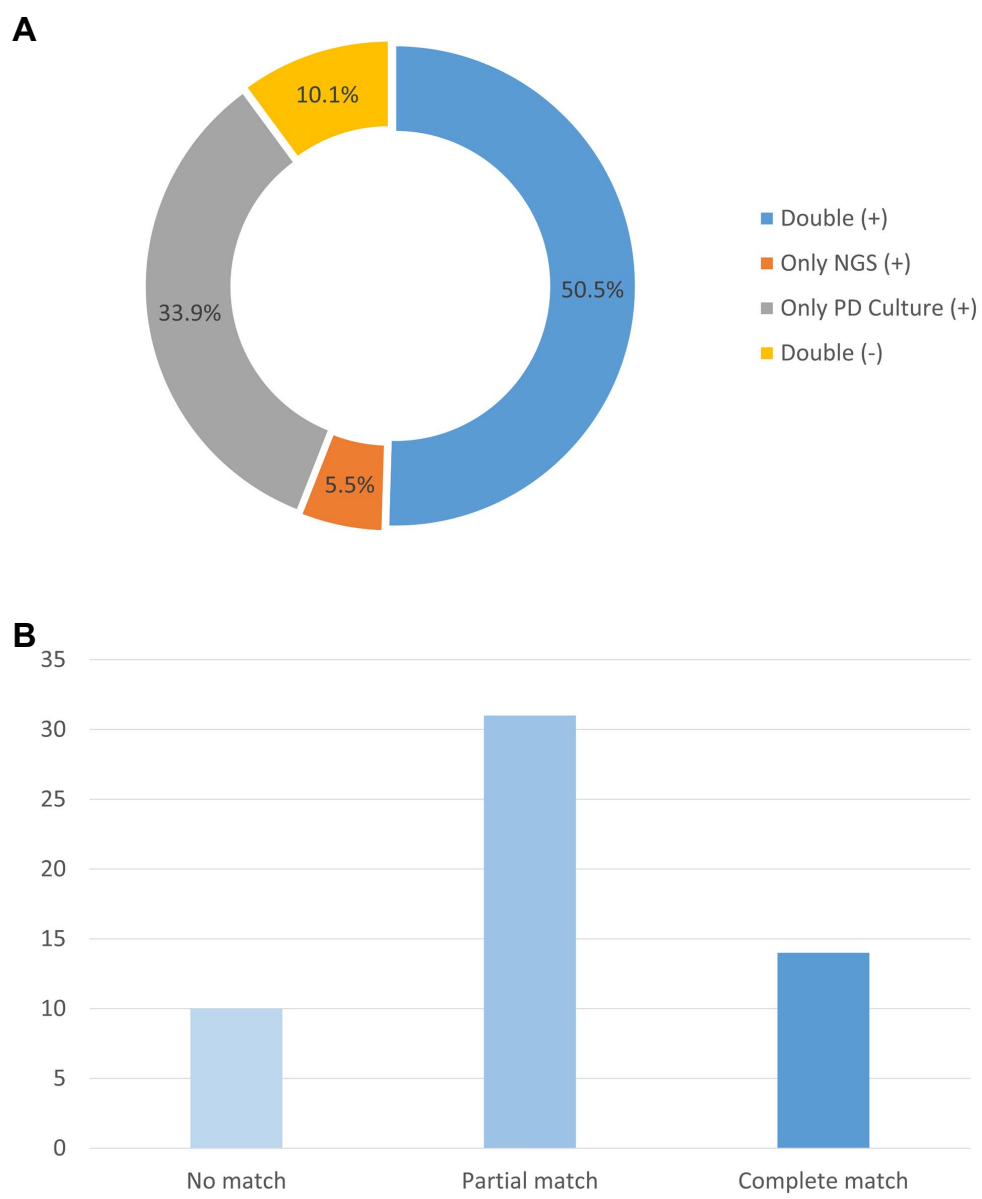
Analysis of the number of samples with matching plasma mNGS and PD culture detection results. **(A)** Proportions of plasma mNGS detection and PD culture results. **(B)** Concordance of the mNGS results for the samples with both positive plasma mNGS and PD culture results. *Y*-axis represents the counts of the patients. mNGS (+), only positive detection in plasma mNGS method; PD Culture (+), only positive detection in PD culture method; Double (+), positive detection for both plasma mNGS and PD culture; and Double (−), negative detection for both plasma mNGS and PD culture.

In the enrolled cohort, 45 patients (41.3%) had at least one matched pair of plasma mNGS and PD culture results, including 31 cases (28.4%) of a partial match, which means at least one pathogen overlapped, and 14 cases (12.8%) of a complete match, as shown in Figure 3. Based on the pathogenic strains, the match analysis showed that approximately half of the plasma mNGS results (in the detected strains) matched those of the corresponding PD culture results for bacterial pathogens (Gram-positive: 18/33; Gram-negative: 42/86), while no matches were observed for fungal pathogens (Fungi: 0/4), as shown in Figure 2. Besides, in the enrolled cohort, 11 patients were diagnosed with concomitant bloodstream infection according to the peripheral blood culture at ICU admission (Supplementary Table 1). Among them, eight cases (72.7%) were positive in mNGS detection; the mNGS results of seven cases completely matched with those of blood culture; and one case partially matched.

We also compared the read counts (rpM) between pathogens. The results showed that the read count for Gram-negative bacteria [19 (7, 92)] was higher than that for Gram-positive bacteria [10 (4, 32), *p* < 0.005] and fungi [2 (1, 59), *p* < 0.005]. Among the Gram-negative bacteria, the read count for *Pseudomonas aeruginosa* [253 (12, 703)] was elevated compared with that of *Klebsiella* spp. [42 (9, 170), *p* < 0.005] and *Escherichia coli* [19 (5, 107), *p* < 0.005], as shown in Supplementary Table 2.

### Clinical Interpretation of mNGS and Comparison With Blood Culture and Smear

**Figure 4:**
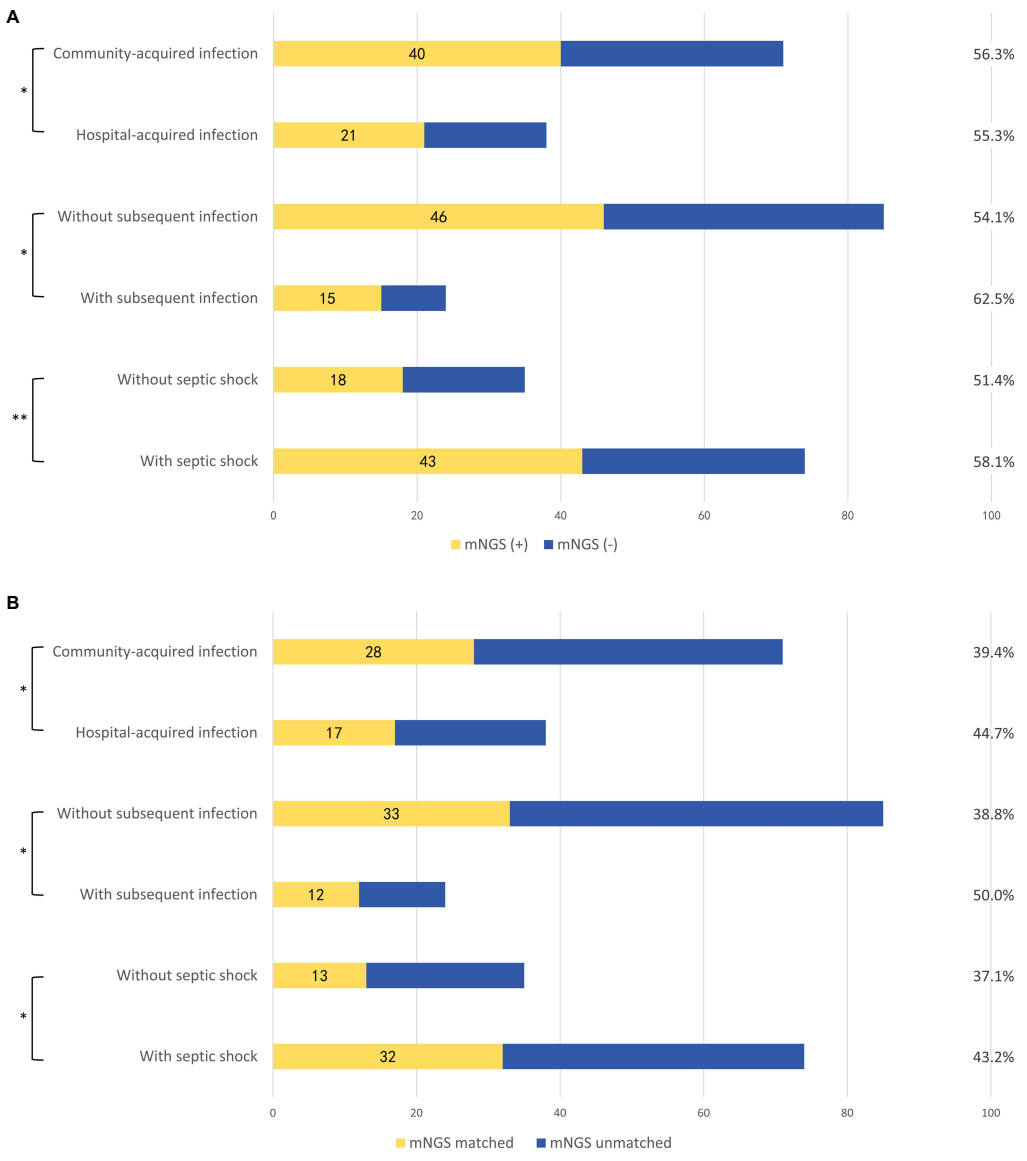
Subgroup analysis according to different clinical scenarios. **(A)** Proportions of plasma mNGS positive samples in different clinical scenarios. **(B)** Concordance of the plasma mNGS detection and PD culture in different clinical scenarios. mNGS (+), positive detection in plasma mNGS method; mNGS (−), negative detection in plasma mNGS method; mNGS matched, the plasma mNGS detection partially or completely was in accord with the PD culture result; and mNGS unmatched, the plasma mNGS detection completely disaccord with the PD culture result. Subsequent infections included: subsequent bloodstream infection, surgical site infection/skin soft-tissue infection, and intestinal fistula. ^*^p < 0.005 and ^**^p = 0.059.

To further interpretate the clinical significance of the mNGS detection, subgroup analysis based on multiple clinical characteristics was performed on positive mNGS and PD culture/mNGS-matched results (Figure 4). Patients with septic shock (58.1 vs. 51.4%, *p* = 0.03) and subsequent infection (62.5 vs. 54.1%, *p* = 0.02) had higher proportions of positive plasma mNGS results than those without septic shock or subsequent infection, respectively. The proportion of matched plasma mNGS and PD culture results was also higher in patients with subsequent infection (41.7 vs. 28.2%, *p* < 0.005) and those with septic shock (43.2 vs. 37.1%, *p* < 0.005), compared with those with no subsequent infection and no septic shock, respectively. The difference of plasma mNGS (+) proportions between patients with community- vs. hospital-acquired infections was not significant (56.3 vs. 55.3%, *p* = 0.059), while the matched proportion of those with hospital-acquired infections was higher (39.4 vs. 44.7%, *p* < 0.005) compared with the community-acquired patients. Besides, the comparison between the clinical outcomes and whether the antibiotics applied matched with the mNGS detection (grouped by Gram-staining positive and negative bacteria) was undertaken. The results showed that for both Gram (+) and Gram (−) bacteria, the patients applied with the mNGS matched antibiotics treatment were associated with statistically insignificant lower hospital mortality [Gram (+): 25.0 vs. 33.3%, *p* = 0.704; Gram (−): 28.6 vs. 33.3%, *p* = 0.981] as well as the ICU stay time and vasoactive usage duration, as showed in Table 2.

**Table 2:** Clinical outcomes of the patients with mNGS positive according to whether received the mNGS matched antibiotics during the first 48 h after ICU admission.

	mNGS positive on Gram (+)			mNGS positive on Gram (−)		
Antibiotics applied accordingly during the first 48 h after ICU admission	Yes	No	*p*	Yes	No	*p*
*n*	16	15		28	15	
Hospital mortality (*n*, %)	4 (25.0%)	5 (33.3%)	0.704	8 (28.6%)	5 (33.3%)	0.981
ICU stay time, hours	198 ± 33	224 ± 56	<0.05	201 ± 38	232 ± 47	<0.05
Vasoactive usage duration, hours	163 ± 28	192 ± 33	<0.05	188 ± 25	213 ± 33	<0.05

Gram (−), Gram-negative bacteria; Gram (+), Gram-positive bacteria.

**Figure 5:**
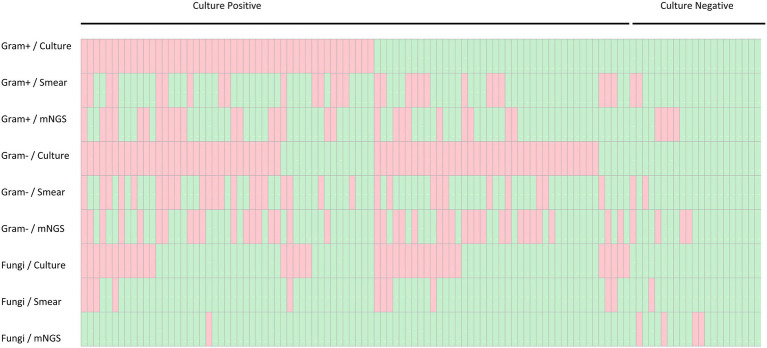
Heatmap depicting plasma mNGS, peritoneal drainage culture and Gram stain smear results stratified by culture result and pathogen category. Gram−, Gram-negative bacteria; Gram+, Gram-positive bacteria.

**Figure 6:**
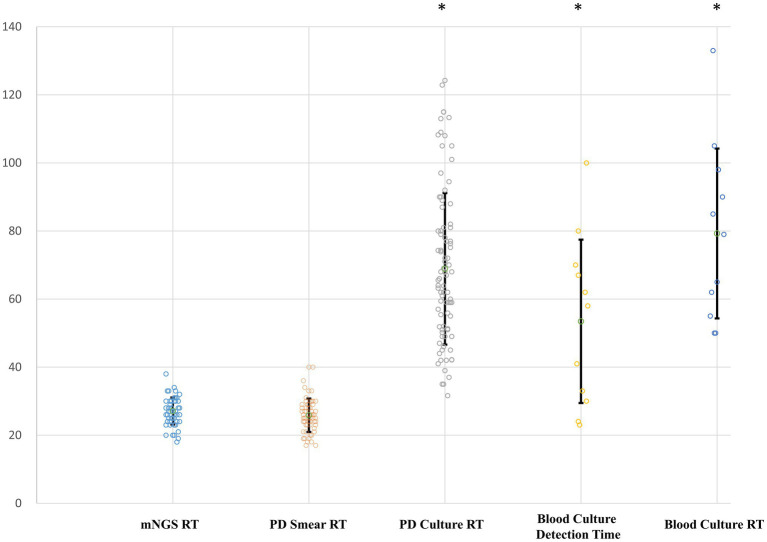
Reporting time (RT) of different pathogen detection methods. *Y*-axis, hours. RT, reporting time from sampling; PD, peritoneal drainage. Differences between the mNGS group and groups with asterisk were considered statistically significant at *p* < 0.05.

Using the PD culture results and different pathogen categories, we evaluated the test performance of plasma mNGS with that of the rapid smear test (Gram staining), and the results are shown in Figure 5. Compared with the conventional smear method, the sensitivity of mNGS in prediction of positive PD culture was superior (Gram-positive bacteria: 18/47 cases of mNGS vs. 15/47 cases of smear, *p* < 0.005; Gram-negative bacteria: 35/68 cases of mNGS vs. 26/68 cases of smear, *p* < 0.005), while the combined method (either plasma mNGS or Gram staining smear) yielded a complementary result (Gram-positive: 26/47, 55.3%; Gram-negative: 47/68, 69.1%). We also compared the reporting time (RT) of mNGS and other methods, as showed in Figure 6. The results showed that, compared with the mNGS reporting time (27.1 ± 4.0 h), the levels of the PD culture reporting time (68.9 ± 22.3 h), the blood culture detection time (53.5 ± 24.0 h), and blood culture reporting time (79.3 ± 25.0 h) all showed statistically significant differences (*p* < 0.05), while the difference between mNGS and PD smear (25.9 ± 4.9 h, *p* = 0.12) did not.

## Discussion

Abdominal sepsis represents a major issue in the management of critically ill patients and is associated with high mortality and morbidity rates (Menichetti and Sganga, 2009; Montravers et al., 2009; Waele et al., 2014). The current literature highlights the importance of the early identification of pathogens and of immediate treatment and microbiological culture. However, the conventional culture process requires up to 48–72 h for a detailed analysis, meaning the selection of appropriate empiric antibiotic therapy can be delayed (Sartelli, 2020). Reportedly, less than 70% of ICU patients with abdominal infections receive appropriate microbiological cultures that can drive the optimization of targeted treatment (Waele et al., 2014). In clinical practice, a novel method is urgently needed for the rapid detection of a wide range of pathogens. In recent years, a new nucleic acid-based method, mNGS, has shown superior feasibility and sensitivity in detecting pathogens and has been validated for use in numerous clinical scenarios, including diagnosing bloodstream infections (Goggin et al., 2019), tuberculosis (Shi et al., 2020), pneumonia (Long et al., 2016), sepsis (Blauwkamp et al., 2019), and pediatric infections (Rossoff et al., 2019). In contrast to blood culture, mNGS analyses of bloodstream infections provide a valuable, non-invasive diagnostic platform for the rapid and early identification of clinically relevant pathogens with higher sensitivity and specificity. However, studies comparing the microbiological diagnostic utility of plasma mNGS with conventional PD culture microbiological methods are still lacking, especially for abdominal infections.

In this study, we enrolled a cohort of 109 patients with abdominal sepsis, in which plasma mNGS detected 61 positive cases while deeming the others to be negative. We compared the demographic and clinical characteristics between the two groups and analyzed the distribution of mNGS and PD culture results and their matched results according to the detected strains or individual cases. The read counts from the mNGS were compared among the different pathogen strains, and subgroup analysis and clinical outcomes comparison were also performed to interpretate the mNGS significance under different clinical scenarios. To our knowledge, this is the first study to describe the application of plasma mNGS in abdominal sepsis patients and to compare its comprehensive microbiological diagnostic performance with that of conventional PD microbiological testing.

The comparison of the clinical characteristics showed that the plasma mNGS positive cases tended to be associated with the etiologies of intestinal perforation or obstruction, a greater number of subsequent infections, and a higher level of hospital mortality. Considering that severe abdominal infection may lead to subsequent bloodstream infection and a worse prognosis, a higher positive rate seems reasonable. The match analysis showed that approximately half of the plasma mNGS results could match those of the corresponding PD culture results for bacterial pathogens. Based on the detected pathogenic strains, the PD culture and plasma mNGS results indicated that most pathogens were Gram-negative bacteria, followed by Gram-positive bacteria, and mNGS had the tendency to detect more Gram-negative bacteria. In contrast to the worse prognosis of the patients with mNGS (+), the clinical outcomes of the patients received antibiotic treatment matched with the mNGS detection during the first 48 h after ICU admission were superior to those did not, which may indicate the potential superiority of mNGS in guide the treatment, and is of great significance for early and appropriate antimicrobial therapy in patients with abdominal sepsis. It was also noteworthy that the plasma mNGS method identified all 10 strains of anaerobic bacteria, which is an important category of pathogens in IAI (Ross et al., 2018).

The fungal infection results were surprising, as mNGS detected only one strain of *Candida*, which is a clinically important abdominal infection pathogen (Xiong and Rao, 2020), while PD culture detected 39 strains. Because of the limited information on its prevalence and few large-scale studies of invasive *Candida* infections, we cannot provide an exact cause of this mismatch between the plasma mNGS and PD culture results. After a comprehensive literature review, we hypothesized that cfDNA released into plasma by fungal is much fewer than that by bacteria in this study condition, which partly implies the specific feature of abdominal infection and is necessary to study further (Goldschmidt et al., 2014; Scharf et al., 2020; Menu et al., 2021). We propose that the mismatch between the mNGS and culture methods in our findings highlights the importance of reconfirming the effects of preanalytical procedures.

Owing to the rapid reporting time, plasma mNGS enables clinicians to obtain an etiological diagnosis within 48 h, while the average conventional PD culture report often requires more than 72 h, the fungal culture may cost even more. In our study, we compared the plasma mNGS findings with those from the widely used smear Gram staining method, and the results showed that, the two rapid methods provided complementary results while the plasma mNGS provided more specific strain identification. In the enrolled cohort, the bloodstream infection was only detected in 11 patients, which was far below the positive cases of mNGS, while the mNGS matched eight cases of the patients with bloodstream infection. The comparison of the reporting time among mNGS and other methods further highlights its advantage of rapid response. It is also noteworthy that mNGS detection was based on nucleic acid sequencing of the peripheral blood, and any other sites of infection besides the peritoneal cavity may lead to positive mNGS results. Positive plasma mNGS results may be associated with more serious cases and each mNGS result should be interpreted according to the individual clinical scenario, as shown in our subgroup analysis.

Previous studies have reported that dynamic changes in plasma mNGS read counts may correlate with the clinical manifestation of diseases and laboratory variables (Ai et al., 2017; Goggin et al., 2019; Zhang et al., 2020). In our study, we compared the read count results according to the different pathogens and discovered that the difference in the number of Gram-positive and Gram-negative bacteria corresponded with the detection distribution of the two categories. This may be because Gram-negative bacteria were the main causes of IAI, and the greater numbers of microorganisms led to a positive mNGS report. Accordingly, the semi-quantitative nature of the read counts may reflect pathogen abundances during the treatment course. In future, a series of mNGS analyses may provide physicians with a new, direct monitoring tool for the diagnosis and treatment of infectious diseases.

Despite these promising results, our study had some limitations. First, we enrolled a relatively small observational cohort. Second, the clinical interventions were not chosen based on the results of the mNGS and, therefore, its significance as a guide to therapy could not be evaluated. Third, only peripheral blood was sampled in our study, and a novel mNGS method of detection using body fluids was published recently (Gu et al., 2021). A comparison between the use of the two methods to analyze peritoneal drainage fluid may provide more valuable information. Finally, because the mNGS results may be easily influenced by various factors, the results obtained in our single-center study should be validated and tested before applying to other centers.

## Conclusion

In summary, this single-center study demonstrated that, in abdominal sepsis patients, plasma mNGS can provide early, noninvasive, and rapid diagnosis by identifying circulating DNA from pathogens. Compared with conventional PD culture methods, plasma mNGS was more rapid and had the tendency to detect Gram-negative bacteria over Gram-positive bacteria and fungi. A combination of plasma mNGS and conventional methods could improve etiology diagnosis. Our observational study found the improved clinical outcomes of the patients received initial antibiotic treatment matched with mNGS detection and future studies with a larger cohort will be needed to assess the significance of plasma mNGS detection for enhancing the treatment of abdominal sepsis cases.

## Data Availability Statement

The datasets presented in this study can be found in online repositories. The names of the repository/repositories and accession number(s) can be found at: https://www.ncbi.nlm.nih.gov/, PRJNA749647.

## Ethics Statement

The studies involving human participants were reviewed and approved by The institutional review board of Peking Union Medical College Hospital. The patients/participants provided their written informed consent to participate in this study.

## Author Contributions

DL designed the study and prepared the drafting of this article. NC conceived the study and made final approval of this manuscript. WG and JZ made analysis of all data and helped revise this manuscript. WG and WC contributed to the acquisition of laboratory data, and HW was in charge of acquisition of clinical data. All authors contributed to the article and approved the submitted version.

## Funding

The work was supported by National Natural Science Foundation of China (No. 82072226), Beijing Municipal Science and Technology Commission (No. Z201100005520049), Non-profit Central Research Institute Fund of Chinese Academy of Medical Sciences (No. 2019XK320040), Tibet Natural Science Foundation [No. XZ2019ZR-ZY12(Z)], and Excellence ProGram of Key Clinical Specialty of Beijing in 2020 (No. ZK128001).

## Conflict of Interest

WG is employed by WillingMed Technology (Beijing) Co., Ltd.

The remaining authors declare that the research was conducted in the absence of any commercial or financial relationships that could be construed as a potential conflict of interest.

## Publisher’s Note

All claims expressed in this article are solely those of the authors and do not necessarily represent those of their affiliated organizations, or those of the publisher, the editors and the reviewers. Any product that may be evaluated in this article, or claim that may be made by its manufacturer, is not guaranteed or endorsed by the publisher.

## Footnotes

^1^ http://www.ncbi.nlm.nih.gov/bioproject/749647
