# Supplementary material for: Metagenomic Next-Generation Sequencing for the Microbiological Diagnosis of Abdominal Sepsis Patients
Source: Front Microbiol. 2022 Feb 2;13:816631. doi: 10.3389/fmicb.2022.816631 (PMC8847725; doi:10.3389/fmicb.2022.816631)
Supplement: Supplementary file 1 [file Data_Sheet_1.PDF]

Supplementary Table 1. Detection and comparison among mNGS, blood culture and peritoneal drainage (PD) culture.

Patient ID	mNGS Detection	Blood culture detection time, hours	Blood culture reporting time, hours	Match of mNGS and blood culture	Blood culture detection	Match of mNGS and PD culture	PD culture detection
1	Enterococcus faecium	23	50	Match	Enterococcus faecium	Partial match	Candida albicans, Acinetobacter baumannii, Klebsiella pneumoniae, Enterococcus faecium
2	NA	80	105	No match	Candida parapsilosis	No match	Candida albicans, Acinetobacter baumannii, Escherichia coli, Klebsiella pneumoniae, Proteus mirabilis
3	Pseudomonas aeruginosa, Bacteroides fragilis, Eikenella corrodens, Haemophilus parainfluenzae, Fusobacterium nucleatum, Escherichia coli, Enterococcus avium, Veillonella parvula	30	55	Match	Escherichia coli, Pseudomonas aeruginosa	Partial match	Escherichia coli， Pseudomonas aeruginosa
4	Bacteroides xylanisolvens, Enterococcus avium	100	133	Match	Bacteroides ovale	Partial match	Acinetobacter baumannii, Brucella citrate, Enterococcus faecalis, Enterococcus avium, Citrobacter freundii

5	Enterococcus faecium	67	85	Match	Enterococcus faecium	Match	Enterococcus faecium
6	Escherichia coli, Pseudomonas aeruginosa	24	50	Match	Pseudomonas aeruginosa	Match	Escherichia coli, Pseudomonas aeruginosa
7	Enterobacter hormaechei, Enterococcus faecalis	33	62	Partial match	Pseudomonas aeruginosa, Enterobacter cloacae	Match	Enterococcus faecalis, Enterobacter hormaechei
8	Escherichia coli	58	79	Match	Escherichia coli	Match	Escherichia coli
9	NA	70	98	No match	Enterococcus faecium, Enterococcus faecalis	No match	Enterococcus faecium, Enterococcus faecalis
10	NA	62	90	No match	Candida albicans, Lactobacillus	No match	Candida albicans, Klebsiella pneumoniae, Lactobacillus, Pseudomonas aeruginosa
11	Klebsiella pneumoniae, Shewanella algae, Enterococcus faecalis, Morganella morganii	41	65	Match	Shewanella algae	Partial match	Candida albicans, Acinetobacter baumannii, Escherichia coli, Klebsiella pneumoniae, Shewanella algae

Supplementary Table 2. mNGS read counts for the detected pathogens

Pathogen	mNGS (+) Cases	Read counts
**Bacteria**	118	18 [6, 65]
**Gram-Positive Bacteria**	33	10 [4, 32]
**Enterococcus spp., n (%)**	23	8 [4, 26]
**Streptococcus spp., n (%)**	3	6 [2,73]
**Staphylococcus spp., n (%)**	7	23 [11, 41]
**Gram-Negative Bacteria**	85	19 [7, 92]
**Klebsiella spp., n (%)**	15	42 [9, 170]
**Escherichia coli., n (%)**	16	19 [5, 107]
**Pseudomonas aeruginosa, n (%)**	4	253 [12, 703]
**Acinetobacter baumannii, n (%)**	2	—*
**Enterobacter spp., n (%)**	34	22 [7, 80]
**Other, n (%)**	3	6 [6, 7]
**anaerobes, n (%)**	11	9 [4, 32]
**Fungi**	5	2 [1, 59]
**Candida, n (%)**	1	—**
**Other, n (%)**	4	28 [1,60]

*** Two *Acinetobacter baumannii* strains were detected with mNGS read counts of 73 and 965, while one strain of *Candida* had a count of 1.**
